# Rifampicin-Loaded Mesoporous Silica Nanoparticles for the Treatment of Intracellular Infections

**DOI:** 10.3390/antibiotics8020039

**Published:** 2019-04-11

**Authors:** Santhni Subramaniam, Nicky Thomas, Hanna Gustafsson, Manasi Jambhrunkar, Stephen P. Kidd, Clive A. Prestidge

**Affiliations:** 1 School of Pharmacy and Medical Sciences, University of South Australia, Adelaide, SA 5000, Australia; santhni.subramaniam@mymail.unisa.edu.au (S.S.); nicky.thomas@unisa.edu.au (N.T.); hanna.gustafsson@unisa.edu.au (H.G.); manasi.jambhrunkar@unisa.edu.au (M.J.); 2 ARC Centre of Excellence in Convergent Bio-Nano Science & Technology, University of South Australia, Mawson Lakes, SA 5095, Australia; 3 The Basil Hetzel Institute for Translational Health Research, Woodville, SA 5011, Australia; 4 Research Centre for Infectious Disease, School of Biological Sciences, The University of Adelaide, Adelaide, SA 5000, Australia; stephen.kidd@adelaide.edu.au

**Keywords:** MSNP, intracellular infections, nanoparticles, antibiotics, SCVs, macrophages, rifampicin

## Abstract

Infectious diseases remain a major burden in today’s world, causing high mortality rates and significant economic losses, with >9 million deaths per year predicted by 2030. Invasion of host cells by intracellular bacteria poses treatment challenges due to the poor permeation of antimicrobials into the infected cells. To overcome these limitations, mesoporous silica nanoparticles (MSNP) loaded with the antibiotic rifampicin were investigated as a nanocarrier system for the treatment of intracellular bacterial infection with specific interest in the influence of particle size on treatment efficiency. An intracellular infection model was established using small colony variants (SCV) of *S. aureus* in macrophages to systemically evaluate the efficacy of rifampicin-loaded MSNP against the pathogen as compared to a rifampicin solution. As hypothesized, the superior uptake of MSNP by macrophages resulted in an enhanced treatment efficacy of the encapsulated rifampicin as compared to free antibiotic. This study provides a potential platform to improve the performance of currently available antibiotics against intracellular infections.

## 1. Introduction

Since the discovery of penicillin in 1928, antibiotics have saved millions of lives worldwide [1]. However, once penicillin became commercially available and hence was more widely used, clinically resistant bacteria rapidly evolved in the 1950s [1,2]. Today, fueled by over-prescription and uncontrolled use of antibiotics, antibacterial resistance has increased dramatically, currently costing the US health care system $21 to $34 billion annually [1].

Phagocytosis is one of the most important defense mechanisms in multicellular organisms, responsible for the destruction of bacteria [3,4]. However, some bacterial species have found a niche to survive the process and live inside the host cells, shielded from standard antibacterial drugs. This poses a significant challenge for treatment of intracellular infections since many antibiotics have low permeability into the cells, poor intracellular retention or poor stability in the acidic environment inside the host cells [2,5,6,7]. It is also important to note that the concentration of antibiotics at the target locations is required to be above the minimum inhibitory concentration (MIC) or minimum bactericidal concentration (MBC), to avoid the re-development of antibacterial resistance by exposure to sub-therapeutic concentrations. Despite the large number of antibiotics available, two-thirds of them are ineffective against intracellular infections [8].

Further increasing the challenge is the presence of bacteria as small colony variants (SCVs), which have long been described in literature and have been an area of research interest for their association with intracellular infections [9,10]. As the name suggests, SCVs are morphologically characterized by colonies of bacteria one-tenth the size of their parent strains [10]. The development of SCVs has been described for many strains of bacterial species including clinically relevant pathogens such as *Staphylococcus aureus*, *Pseudomonas aeruginosa* and *Escherichia coli* [10], although most studies have largely focused on SCVs of *S. aureus*. It has been hypothesized that SCVs are one important reason why *S. aureus* infections remain clinically challenging [11].

Novel therapies, such as the use of antimicrobial peptides and antisense oligonucleotides, have been studied for the treatment of intracellular infections. Antimicrobial peptides exhibit bactericidal activity by disrupting bacterial membranes to cause leakage of cellular contents while antisense oligonucleotides silence expression of genes essential for the survival of bacteria [2,12,13]. Phage therapy, i.e., the use of viruses that target specific bacteria, is re-emerging as a potential treatment strategy especially for pulmonary infections [14]. Enzybiotics or bacteriophage cell wall hydrolases are being tested in clinical trials for their antibacterial activity by cleavage of specific cell wall bonds [14].

While some of these novel antimicrobials are promising approaches, they face uncertainties regarding clinical efficacy as well as long and expensive regulatory hurdles. In contrast, significant research has been carried out on harnessing existing antimicrobials by encapsulation in nanoparticles for improved efficacy against infections [15,16,17]. The nanoparticulate drug delivery approach for intracellular infections mimics the endocytic or phagocytic pathway of pathogens to localize within the host cells [8]. Nanotechnology offers unprecedented opportunities for intracellular drug delivery by engineering nanoparticles with various composition and surface functionalization [2]. Moreover, nanotechnology can be employed to achieve sustained drug release, reduce side effects, increase drug efficacy and protect drugs from enzymatic degradation [18].

Liposomes are one of the most widely researched drug delivery vehicles for intracellular infection [8,18]. Studies have shown that upon parenteral administration, liposomes are rapidly taken up by macrophages in the liver and spleen [18]. However, the stability of liposomes after administration remains a major challenge as the vesicles are exposed to destabilizing proteins, such as lipoproteins. Other issues include low drug loading/entrapment and drug leakage compared to other carriers, and also the requirement for sterilization can compromise the integrity of liposomes.

To avoid stability issues exhibited by liposomes, polymeric nanoparticles with a size range of 100–1000 nm have been developed as alternative nanocarriers. They are developed using natural polymers such as starch, alginate, and chitosan or synthetic polymers such as poly(lactic-*co*-glycolic acid) PLGA and poly(lactic acid) (PLA) [18]. Preparation techniques and selection of polymer material highly depends on the physicochemical characteristics of the drug [18]. Copolymers have also been developed to increase drug load and to offer both an immediate and sustained release of drug. PLA and PLGA particles are broken down into molecules that are cleared from the body via metabolic pathways [8,18]. In spite of the fact that polymeric nanoparticles are capable of delivering antibacterial drugs intracellularly, scaling-up of synthesis, sterilization, and residues of solvents typically used in fabrication process remain problematic prior to the translation to broader clinical applications [8].

To overcome these issues, mesoporous silica nanoparticles (MSNP) were investigated as a nanocarrier for effective delivery of antibiotics intracellularly. MSNPs are honeycomb-like porous structures with a large number of pores that are capable of adsorbing or encapsulating relatively large amounts of molecules [19]. Their advantages include: tunable pore sizes, specifically within a range of 2–15 nm, allowing encapsulation of small and large molecules, and that their synthesis is scalable, cost-effective and relatively simple [19,20]. Silica is used in cosmetics and as a food additive and is classified as “Generally Recognized as Safe” (GRAS) by the US Food and Drug Administration (FDA) [20,21]. Silica nanoparticles are biodegradable, thus avoiding issues related to tissue accumulation during long-term use [21,22]. Silica (nano)particles are degraded into orthosilicic acid, a water-soluble compound that humans naturally absorb [21].

Even though MSNPs have been widely researched as a biocompatible drug carrier, most of the research has focused on its use in cancer therapy while only few reports have been published in the area of intracellular infection [23,24,25]. Studies using infection models are necessary to fill this void to understand how the properties of MSNP can influence their uptake by different cells. Moreover, to the best of our knowledge research on the effectiveness of antibiotic-loaded MSNP against SCV-associated intracellular infection has not been reported.

Rifampicin is one of the most efficacious drugs against intracellular *S. aureus* [26,27]. However, its ability to accumulate intracellularly is strongly hindered by its poor solubility and permeability across the gut when given orally [28,29,30] or other biological barriers. Moreover, rifampicin has been reported to have a varied oral bioavailability within the range of 36.6 to 93% [29,30]. When given at very high doses, rifampicin can cause hepatotoxicity, limiting its clinical use [31]. Therefore, rifampicin was chosen as a model drug to test its efficacy against small colony variants (SCV) of *S. aureus* present intracellularly in RAW 264.7 murine macrophages.

Herein, we report the ability of MSNP to deliver rifampicin into macrophages to treat SCV-associated intracellular infections.

## 2. Results and Discussion

### 2.1. Synthesis and Characterization of MSNP

**Figure 1:**
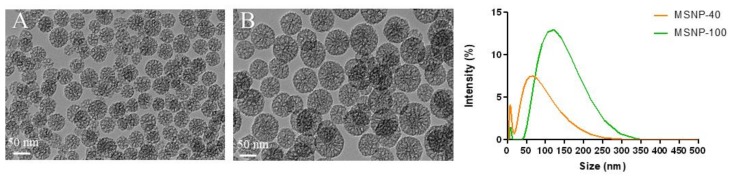
(**Left**) Transmission electron microscopy (TEM) images of (**A**) mesoporous silica nanoparticles with particle sizes of 40 nm (MSNP-40) and (**B**) mesoporous silica nanoparticles with particle sizes of 100 nm (MSNP-100). (**Right**) Particle size distribution of MSNP-40 and MSNP-100 dispersed in Dubelcco’s modified eagle medium (DMEM) as measured by dynamic light scattering (DLS). The peak at 10 nm represents the fetal bovine serum (FBS) present in DMEM [34].

Hiroshima mesoporous material (HMM)-type mesoporous silica nanoparticles with particle sizes of 40 nm (MSNP-40) and 100 nm (MSNP-100) were successfully synthesized. Transmission electron microscopy (TEM) images (Figure 1) confirmed spherical nanoparticles of 40 nm and 100 nm with a non-ordered porous structure.

While many preparation methods for mesoporous silica nanoparticles have been described, including for the common mobile composition of matter (MCM) and Santa Barbara amorphous (SBA)-type MSNPs, these methods are less suitable for the controlled synthesis of spherical particles with a particle size smaller than 100 nm [32]. Furthermore, the pore diameters of MCM and SBA-type particles are typically <5 nm, which can limit their ability to load larger drug molecules via passive adsorption thereby, reducing drug loading capacity [32,33].

Two methods were used to determine the size of particles, (1) analysis based on TEM images and (2) dynamic light scattering (DLS) measurement. The summary of the results from both size measurements and the zeta potential is presented in Table 1.

For DLS measurements, DMEM was used as a dispersant as this provides a more accurate representation of the environment the particles are exposed to in the cellular studies. The removal of aggregated nanoparticles by centrifugation yielded nanoparticles of desired sizes with a relatively narrow particle size distribution. It should be noted that due to the presence of FBS (peak around 10 nm), the calculated z-average and polydispersity index (PDI) do not accurately reflect the true size of the nanoparticles and corresponding PDI. Moreover, since DLS measures the hydrodynamic diameter of particles rather than the size of dehydrated particles, the particle sizes obtained from DLS measurements were typically compared to TEM.

Based on image analysis using Fiji (Fiji is Just ImageJ 1.52h) [35], the pore sizes of both nanoparticles were between 8 and 9 nm. Brunauer–Emmett–Teller (BET) particles surface analysis was not performed as in a dry state, the intercalated cavities of the aggregated nanoparticles are also measured, thereby not providing an accurate measurement of pore size and surface area.

**Figure 2:**
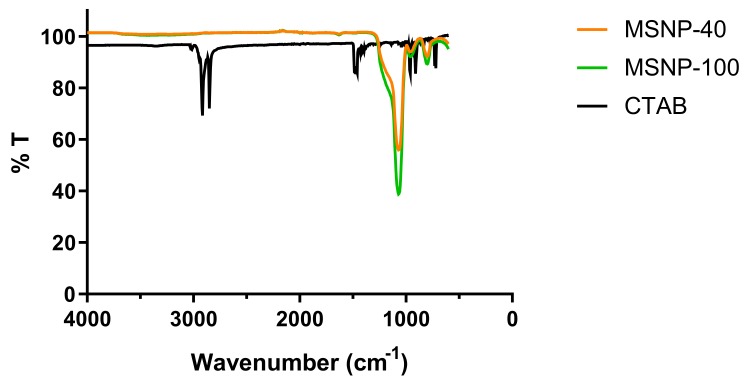
Fourier transform infrared (FTIR) spectra of MSNP-40, MSNP-100, and cetyltrimethylammonium bromide (CTAB) following solvent extraction.

Due to reported toxicity of cetyltrimethylammonium bromide (CTAB), attenuated total reflection-Fourier transform infrared (ATR-FTIR) analysis was performed to confirm the removal of the surfactant template from the nanoparticles. As illustrated in the IR spectra (Figure 2), CTAB shows two peaks at approximately 2800 cm^−1^ corresponding to the C-H stretching of the methylene groups (adjacent to the quaternary ammonium cation) in CTAB [36]. The absence of these peaks in the synthesized particles indicated the successful removal of CTAB from the nanoparticles.

### 2.2. Cytotoxicity Studies

**Figure 3:**
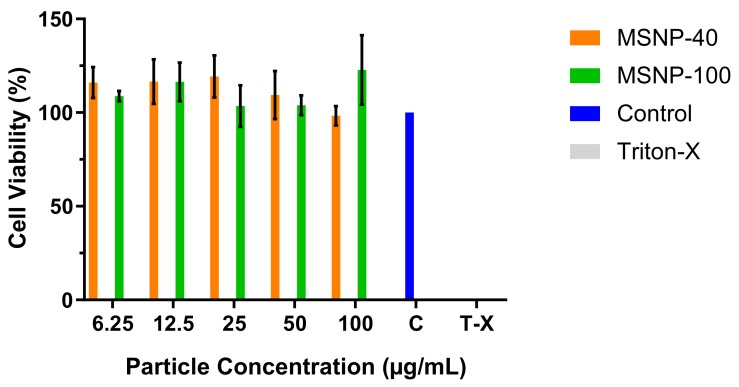
Cellular viability as determined via MTT assay (mean ± SD, *n* = 3).

Cellular viability following exposure to MSNP was quantified using MTT ((3-(4,5-dimethylthiazol-2-yl)-2,5-diphenyltetrazolium bromide) assay to establish potential cytotoxicity of the engineered particles. MSNPs were not cytotoxic over the concentration range investigated (6.25 to 100 μg/mL) since the cellular viability was well above 80%, the commonly accepted threshold for cell viability and, therefore, suitable for the subsequent cell uptake studies [37] (Figure 3). Triton-X was added as a negative control at a concentration of 25 µg/mL where no cellular viability was detected (note: results are not shown in the graph as value is too small).

### 2.3. Cellular Uptake of MSNP

**Figure 4:**
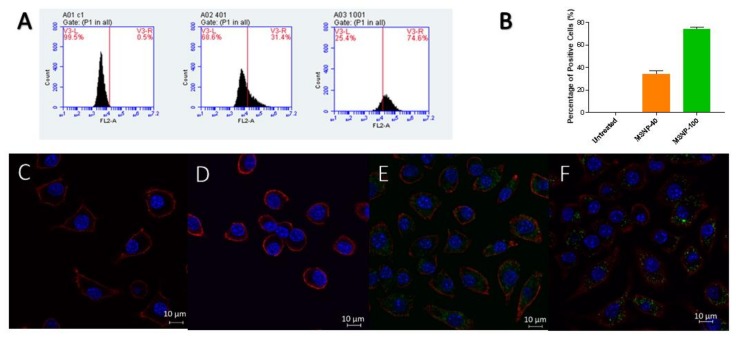
(**A**) Raw data obtained from fluorescence-activated cell sorter (FACS) indicating the shift in fluorescence intensity (Note: Graphs shown are only from one sample). C1 = untreated, 401 = MSNP-40, 1001 = MSNP-100. (**B**) Uptake of rhodamine-loaded MSNP quantified by FACS ((mean ± SD, *n* = 3). (*p* < 0.05 for MSNP-40 compared to MSNP-100). (**C**–**F**) Laser scanning confocal microscopy (LCMS) images of (**C**,**D**) untreated cells, (**E**) MSNP-40 and (**F**) MSNP-100. Nuclei were stained with DAPI (Blue), MSNP were stained with rhodamine (Green) and the cell cytoskeleton was stained with wheat germ agglutinin—Alexa fluor 633 (Red).

Two methods were employed to determine the uptake of MSNP in RAW 264.7 cells, (1) Fluorescence-activated cell sorter (FACS) for quantitative and (2) laser scanning confocal microscopy (LCMS) for qualitative assessment (fluorescent dye rhodamine B was used as a model drug). As exemplified in Figure 4, the raw data showed a shift in fluorescence intensity as compared to untreated cells, which translated to the internalization of dye-loaded MSNP by RAW 264.7 cells.

Quantification of the particle uptake by FACS (Figure 4) indicated remarkable differences in the cellular uptake, depending on the size of MSNP. MSNP-100 were taken up at significantly higher levels than MSNP-40. This difference in uptake between MSNP-40 and MSNP-100 can be attributed to the uptake mechanism of the macrophages, either via phagocytosis, macro- or micropinocytosis or endocytosis [38]. There are two mechanisms for endocytosis, clathrin-mediated and caveolin-mediated endocytosis. Clathrin-mediated endocytosis is a receptor-mediated process where materials are transported into the cells by forming vesicles of 60 to 200 nm (also referred to as clathrin-coated pits) that fuse with lysosomes [39]. Caveolae exist in cholesterol-rich regions of the plasma membranes and forms invaginations of 50–100 nm for internalization of materials [39,40].

Both mechanisms have been previously proposed to be affected by the size and type of nanoparticles on cellular uptake. Previous studies have reported multiple uptake mechanisms involving clathrin-mediated and micropinocytosis or phagocytosis for 40 nm polystyrene nanoparticles in J774A.1 murine macrophage [38]. Another study has reported silica nanoparticles uptake in THP-1 human macrophages via clathrin-mediated endocytosis, in addition to caveolin-mediated endocytosis for particle sizes of 60 nm and 200 nm [39]. Furthermore, following rapid uptake of nanoparticles by macrophages, nanoparticles with a smaller size have been reported to show high levels of exocytosis [41]. Chithrani et al. have also reported exocytosis to be a size-dependent process in HeLa cell lines, with smaller particles displaying higher rates of exocytosis—which concept may be similar to the above observation [42]. Exocytosis is defined as the process in which the contents in a secretory vacuole are released to the external environment by fusion of the vacuole membrane with the cell membrane [43]. This could also be a possible explanation for the lower cellular uptake observed for MSNP-40 as compared to MSNP-100.

### 2.4. Loading Capacity and Encapsulation Efficiency of Rifampicin

Rifampicin was successfully loaded into MSNP via passive diffusion method using a concentration ratio of 2:1 (drug to MSNP). Methanol was chosen as the solvent as rifampicin is freely soluble in methanol and the previous study showed methanol to offer a higher drug entrapment efficiency as compared to DMSO [44].

Upon loading, MSNP encapsulated with rifampicin (MSNP-Rif) were centrifuged to remove free rifampicin, washed thrice with its supernatant analyzed for free rifampicin, determined using UV-vis spectroscopy. The loading capacities and encapsulation efficiencies of rifampicin in MSNP (MSNP-Rif) are summarized in Table 2.

### 2.5. In Vitro Release Study

**Figure 5:**
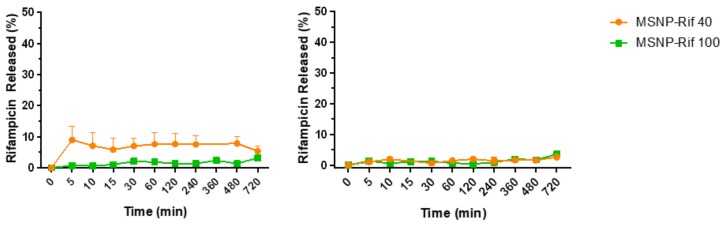
Release kinetics of rifampicin from (**left**) MSNP in phosphate buffer solution (PBS) (pH 7.4) and (**right**) acetate buffer (pH 5.0) (mean ± SD, *n* = 3). Note: error bars are smaller than symbols.

The in vitro release of rifampicin from MSNP (Figure 5) was quantified under two different conditions, (1) PBS buffer at pH 7.4 and (2) acetate buffer at pH 5.0, under sink conditions at 37 °C. These conditions were selected to resemble the physiological conditions of the body and intracellular environment of infected macrophages, respectively. Samples were collected at predetermined time points, diluted with methanol and analyzed using UV-vis spectroscopy.

At pH 7.4, approximately 10% of rifampicin were released within 5 min from MSNP-Rif 40 while only 3% were released from the larger MSNP-Rif 100. This is likely attributed to the larger surface area of the smaller particles and rifampicin attached on the surface of MSNP. In contrast, the release of rifampicin at pH 5.0 was comparable for both MSNP-Rif 40 and MSNP-Rif 100 which can likely be attributed to the low solubility of rifampicin at more acidic conditions compared to pH 7.4 [45]. No changes in release profile were observed for a period within 12 h.

The high hydrophobicity of rifampicin can likely be attributed to the limited drug release from MSNP in both conditions. This is in line with a previous study, where DMSO was used to elute rifampicin from MSNP completely due to limited release in pH 7.4 and pH 5.0 buffers [23]. As mentioned previously, DMSO does not represent physiological conditions but may be chosen to mimic the hydrophobic environments of macrophages [23].

### 2.6. Minimum Inhibitory Concentration (MIC) and Minimum Bactericidal Concertation (MBC) of Rifampicin in SCV S. aureus

The concentration of rifampicin used for the intracellular efficacy study was based on the MIC and MBC against an SCV strain of *S. aureus* and clinical considerations. A standard oral dose of 600 mg of commercially available rifampicin results in peak serum concentration between 4 to 32 µg/mL in healthy adults [46]. The experimentally determined MIC and MBC against SCV *S. aureus* were 0.125 µg/mL and 4 µg/mL, respectively. A rifampicin concentration four times higher (0.50 µg/mL) than the determined MIC, was selected as the concentration for the intracellular infection assay. Assuming uptake of the drug-loaded particles by macrophages in vitro, the inhibition of the intracellular growth of bacteria would be expected at a far lower concentration than a typical clinical dose.

### 2.7. Efficacy of Rifampicin-Loaded MSNP against Intracellular SCV S. aureus

**Figure 6:**
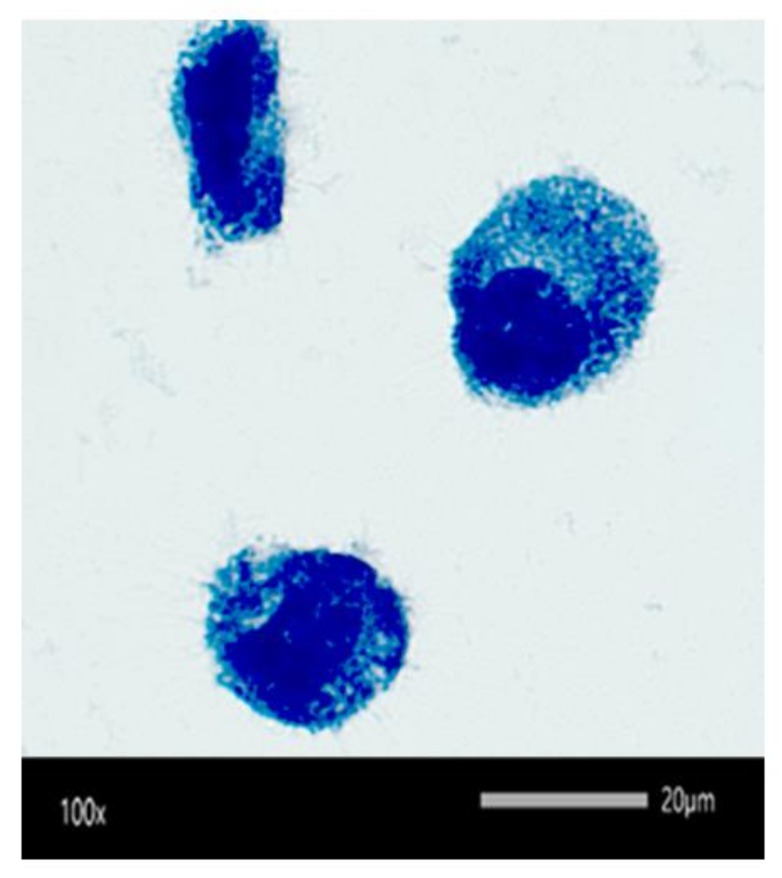
The intracellular presence of small colony variants (SCV) *S. aureus* (stained dark blue) as observed under the light microscope using Leishman’s staining.

The internalization of SCV *S. aureus* into RAW 264.7 macrophages was visually confirmed by Leishman’s staining and light microscopy (Figure 6). The dark blue spots resembled SCV *S. aureus*, the light blue region represented the cytoplasm of the cell while the large dark blue circles represented the nucleus of the cell.

By using MSNP as a nanocarrier for rifampicin, it was hypothesized that a sufficiently high intracellular concentration of rifampicin will be obtained, translating to better efficacy of rifampicin activity compared to unformulated rifampicin.

**Figure 7:**
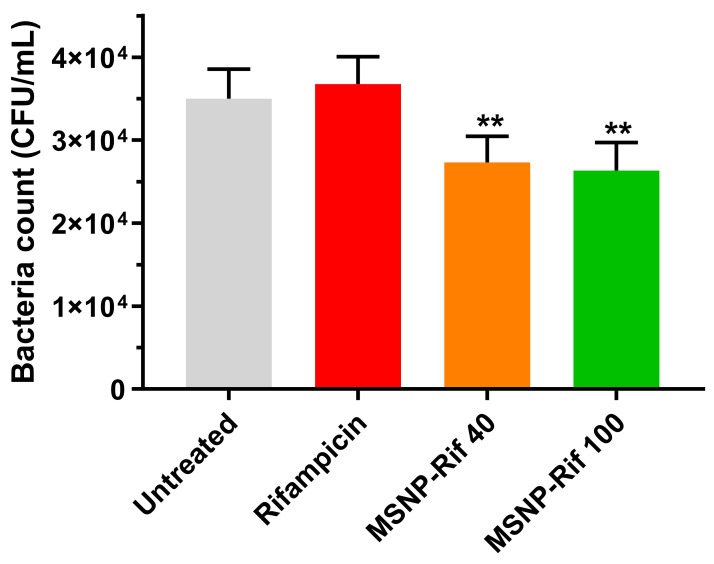
Efficacy of rifampicin and MSNP-Rif against SCV *S*. *aureus* (mean ± SD, *n* = 3). (Note: ** indicates MSNP-40 and MSNP-100 significantly different to untreated and unformulated rifampicin, *p* < 0.05).

Indeed, both MSNP-Rif 40 and MSNP-Rif 100 significantly enhanced the antibacterial activity compared to unformulated rifampicin (Figure 7), (*p* < 0.05) after 4 h exposure to the treatments. At a concentration of 0.50 μg/mL, free rifampicin demonstrated no antibacterial activity against SCV *S. aureus* compared to untreated control (*p* > 0.05), which can be attributed to the poor penetration and uptake of free rifampicin by macrophages. In contrast, at the corresponding concentration of rifampicin, loaded in MSNP, particle uptake by macrophages translated to enhanced antibacterial activity.

Despite the differences in cellular uptake of MSNP-40 and MSNP-100 as quantified by FACS, no statistically significant difference was observed on the antibacterial activity of MSNP-Rif 40 and MSNP-Rif 100 (*p* > 0.05). Bouchoucha et al. have reported significantly higher anti-tumoral activity of doxorubicin-loaded MSNP of 45 nm than 150 nm in cancer cell lines [47]. However, possibly attributed to the different uptake mechanism and the intracellular environment in infected macrophage, a similar trend was not observed in this study.

Enhanced antibacterial activity of MSNP-Rif compared to unformulated rifampicin was observed despite minimal release of rifampicin from MSNP. This is in line with recent studies that have demonstrated synergistic effects of antibiotics-loaded MSNP compared to free antibiotic at corresponding concentrations, thereby not requiring a complete release from the nanocarrier system [48,49,50]. Aguilar-Colomer et al. [51] have reported the effective use of rifampicin encapsulated in MSNP against biofilm. While this study was limited to the scope of intracellular infection, it would be of future interest to evaluate the effectiveness of MSNP against both, biofilm and intracellular infection using different antibiotics and bacterial strains.

## 3. Materials and Methods

### 3.1. Materials

Cetyltrimethylammonium bromide (CTAB), tetraethyl orthosilicate (TEOS), L-lysine, phosphate buffer solution (PBS) tablets, sodium chloride (NaCl), sodium acetate and rhodamine B were all purchased from Sigma-Aldrich (Castle Hill, NSW Australia). N-hexane, dimethyl sulfoxide (DMSO), methanol (MeOH), hydrochloric acid (HCl) and acetic acid were sourced from ChemSupply (Gillman, SA Australia).

### 3.2. Fabrication of MSNP

Hiroshima mesoporous material (HMM) mesoporous silica nanoparticles with controllable morphology were prepared as described in the literature with some modifications [52]. CTAB was used as a templating agent, TEOS as a silica source, hexane as the hydrophobic component and lysine as a catalyst. An oil-in-water emulsion was first formed by vigorously stirring 800 mg CTAB, 180 mg L-lysine in 248 g of Milli-Q water and 60 g hexane for one hour at 70 °C. Eight grams of TEOS was subsequently added and stirring was continued for another 20 h at the same temperature. The suspension was cooled to room temperature and dried in the oven for three hours at 80 °C to form a white powder. The template was then removed via solvent extraction, where the powder was resuspended in a mixture of 90 mL of MeOH and 10 mL of 12 M HCl and left for reflux at 70 °C overnight. The suspension was centrifuged at 20,000 rpm for 10 min. After washing with MeOH, the particles were refluxed again to ensure all of the CTAB has been removed. Upon centrifugation and washing, the particles were dried in the oven.

For the synthesis of 100 nm MSNP, a similar procedure was followed with the exception of using 400 mg CTAB, 90 mg L-lysine in 124 g Milli-Q water, 60 g hexane and 4 g of TEOS.

To remove the presence of aggregates from the synthesized nanoparticle dispersion, a purification step was employed via centrifugation. The supernatant containing well-dispersed MSNP were collected and the concentration was determined using UV vis spectroscopy with rhodamine-loaded MSNP (wavelength = 543 nm). The amount of centrifuged MSNP was adjusted accordingly prior to any studies and re-dispersed in desired media.

### 3.3. Characterization of MSNP

To characterize the particle size and morphology, samples at a concentration of 1 mg/mL were dispersed and sonicated in ethanol for an hour. Five microliters of the sample was transferred to TEM copper grids and air-dried. The Fiji (Fiji is Just ImageJ) (ImageJ 1.52h, National Institutes of Health, USA) [35] software was used to measure the particle and pore size of the MSNP.

The particle size of all formulations was analyzed by DLS (Malvern Zetasizer Nano ZS, Worcestershire, UK). Particles were prepared according to the previously described method (Section 3.2) and diluted 1:100 in DMEM (refractive index = 1.345) prior to measurements at 37 °C. To measure the zeta potential, 100 μg/mL of sample was diluted in 10 mM NaCl and measured accordingly.

The infrared spectra of CTAB, 40 nm and 100 nm MSNP were recorded on a Perkin Elmer, Spectrum Two (Waltham, MA, USA) spectrometer using a universal ATR, over a 600–4000 cm^−1^. No sample preparation was required using this instrument.

### 3.4. Rhodamine Loading into MSNP

To load the particles with rhodamine, MSNP were dispersed at a concentration of 1 mg/mL in Milli-Q water via sonication. Rhodamine stock solution of 1 mg/mL in Milli-Q water was added to the particles at a ratio of 1:100 (dye to particles) and left to stir for 2 h. The particles were collected by centrifugation and washed three times with Milli-Q water to separate the loaded particles from free rhodamine.

### 3.5. Cell Culture Conditions

Cellular studies were carried out in murine macrophages (ATCC RAW 264.7) (passage 9–25). Cells were cultured at 37 °C in an incubator supplied with 5% CO_2_ in Dubelcco’s Modified Eagle Medium (DMEM), supplemented with 10% fetal bovine serum (FBS) and 1% penicillin. RAW 264.7 cells were cultured in T25 flasks at a concentration of 1 × 10^5^ cells/cm^2^. Cells were routinely subcultured every four days, upon cells reaching 80–90% confluency.

### 3.6. Cytotoxicity Studies

RAW 264.7 cells were seeded in a 96 well plate at a density of 1 × 10^4^ cells per well an incubated overnight. The cells were treated with MSNP dispersed in DMEM and left to incubate for 24 h. After 24 h, 200 μL of MTT stock solution (5 mg/mL in DMEM) was added and the cells were left to incubate for another 4 h. DMSO was added and incubated on an orbital shaker for 15 min to dissolve MTT formazan. The absorbance was measured at a wavelength of 540 nm. The relative absorbance of treated cells to untreated cells was used as a relative measure of cellular viability. Triton-X was added at a concentration of 25 μg/mL as a negative control.

### 3.7. Flow Cytometric Analysis (FACS)

To evaluate the uptake of particles, RAW 264.7 cells were seeded at a cell density of 1 × 10^5^ cells per well in a 48 well plate. The cells were grown for 24 h at 37 °C and 5% CO_2_. Rhodamine-loaded MSNP were dispersed in 1 mL of serum-free media which was further diluted to give a final concentration of 50 μg/mL rhodamine-loaded MSNP. Following the removal of cell culture medium, fresh serum-free media (1 mL) containing 50 μg of rhodamine-loaded MSNP was added to the cells and left to incubate for 4 h (37 °C, 5% CO_2_). After media removal, the cells were washed thrice with sterile PBS. The cells were gently harvested from the bottom of the well and transferred to Eppendorf tubes by gentle scraping. The cells were centrifuged at 280 rcf for 5 min to collect the cell pellet. The supernatant was discarded and replaced with 200 μL of FACS buffer. Prior to analysis, the cells were dispersed by gentle mixing using a pipette. The percentage of positive cells (percentage uptake) was determined by the shift in the mean fluorescence intensity compared to the mean fluorescence of untreated cells (set as the threshold).

### 3.8. Confocal Imaging

Cells were seeded on coverslips in a six well plate at a cell density of 1 × 10^5^ cells per well and left to incubate for 24 h (37 °C, 5% CO_2_). Fifty micrograms per milliliter of rhodamine labeled MSNP in serum-free media were added to the cells and incubated in the same condition for 4 h. The wells were then washed with PBS (supplemented with 1% FBS) three times. After washing, 500 μL of a 4% formaldehyde solution was added to fix the cells and left in the fridge overnight. The following day, the formaldehyde solution was removed, and the cells were washed with PBS thrice. To stain the cytoskeleton of cells, 5 μL of PBS containing 1 mg/mL of WGA, Alexa Fluor^®^ 633 conjugate (Invitrogen, Carlsbad, CA, USA) was added to the coverslips and left to incubate for 5 min. Cells were washed with PBS and 1 mL of absolute ethanol was added to the cells and incubated at room temperature for 30 min. Cells were washed again with PBS. Five hundred microliters of bovine serum albumin (BSA) solution was added and cells were further incubated for 30 min at room temperature. After washing, one drop of 4′,6-diamidino-2-phenylindole (DAPI) dye was added to a clean glass slide, and the coverslip containing stained cells were fixed unto the slide. Once slides were dry, cells were imaged using LMS 710 Carl Zeiss confocal microscopy (Oberkochen, Germany).

Laser excitation at 453 nm, 518 nm, and 646 nm were used for samples stained with WGA, Alexa Fluor^®^ 633 conjugate, rhodamine B and DAPI respectively. ZEN Lite 2.6 software was used for image processing.

### 3.9. Rifampicin Loading into MSNP

The loading of rifampicin into the MSNP was carried out according to a previously published method [44]. Briefly, 2 mg of particles were added to 2 mg/mL of rifampicin in methanol (1:2 particles to drug ratio). The suspensions were stirred at 500 rpm for 24 h. The particles were then centrifuged, washed and dried overnight in an oven at 60 °C. Recovered (non-encapsulated) rifampicin in the supernatant obtained from washed rifampicin-loaded MSNP was quantified by UV-vis spectroscopy (wavelength = 254 nm). The loading capacity and encapsulation efficiency were calculated using Equation (1) (loading capacity (%) of Rif-MSNP) and Equation (2) (encapsulation efficiency (%) of Rif-MSNP).

(1) $$\mathrm{Loading}\;\mathrm{Capacity}\;\left(\%\right)=\;\frac{\mathrm{Initial}\;\mathrm{Rifampicin}\;\left(\mathrm{mg}\right)-\mathrm{Recovered}\;\mathrm{Rifampicin}\;\left(\mathrm{mg}\right)}{\mathrm{Mass}\;\mathrm{of}\;\mathrm{MSNP}\;\left(\mathrm{mg}\right)}\;\times 100$$

(2) $$Encapsulation\;Efficiency\;\left(\%\right)=\;\frac{Initial\;Rifampicin\;\left(mg\right)-Recovered\;Rifampicin\;\left(mg\right)}{Initial\;Rifampcin\;\left(mg\right)}\;\times 100$$

### 3.10. In Vitro Release of Rifampicin

In vitro release of rifampicin was investigated for 40 nm and 100 nm loaded MSNP in PBS (pH 7.4) and acetate buffer (pH 5.0). Five milligrams of rifampicin-loaded MSNP were suspended in 10 mL of PBS (sink conditions) and agitated at 37 °C. Samples were withdrawn at pre-determined time points and centrifuged at 38,000 rcf for 10 min. The supernatant was diluted in methanol and quantified for rifampicin by UV-vis spectroscopy as described above.

### 3.11. MIC and MBC of Rifampicin

The MIC was determined by the broth dilution method following standard protocols [53,54]. Briefly, single colonies of *S. aureus* stable SCV blood isolate strain, WCH-SK2 [55] were diluted in 0.9% saline to 1 × 10^8^ colony forming units (CFU)/mL (equivalent to an optical density of 0.08–0.12 at 600 nm). The bacterial suspension was further diluted (1 in 100) with Mueller–Hinton (MH) broth. Bacterial suspension was added to wells containing a serial dilution of rifampicin in 5% DMSO (range 0.03 to 32 µg/mL). Following incubation for 24–30 h at 37 °C, the plates were visually assessed for turbidity indicating growth of bacteria. The lowest concentration of rifampicin that did not promote bacterial growth was defined as the MIC.

A loop from each well of the overnight MIC plates (post-treatment) was transferred to sterile tryptic soy agar (TSA) plates and incubated for 16 to 20 h at 37 °C. The lowest concentration at which no visual growth of bacterial colonies was observed indicated the MBC.

### 3.12. Intracellular Infection Assay

The intracellular infection model was established by modifying previously reported procedures [23,24].

RAW 264.7 cells were plated at a cell density of 1 × 10^4^ in a 48 well plate and left to incubate for 24 h (37 °C, 5% CO_2_). An overnight culture of SCV *S. aureus* was prepared in tryptic soy broth (TSB). Bacterial overnight culture was prepared to a multiplicity of infection (MOI) of 10:1 and redispersed in cell media (DMEM inoculum) upon collection of the bacterial pellet via centrifugation. The cells were incubated with 1 mL of DMEM inoculum for 1 h (37 °C, 5% CO_2_). Thereafter, the cells were washed thrice with sterile PBS to remove the presence of extracellular bacteria. One milliliter of serum-free media containing treatment was added to the cells. Rifampicin (dissolved in 5% DMSO) was added at concentrations corresponding to MSNP-Rif 40 and MSNP-Rif 100. Positive controls wells were filled with only serum-free media. After 4 h, the media was removed and replaced with 1 mL of 0.1% Triton-X in PBS to lyse the cells and extract the bacteria. The cells were transferred to Eppendorf tubes, serially diluted with sterile saline, spot plated on TSA plates (20 µL) and incubated for 18 to 24 h at 37 °C. Serial dilutions of the DMEM inoculum were also performed and plated on TSA plates to confirm the number of bacteria in the inoculum.

To visualize the presence of intracellular bacteria, Leishman’s staining was conducted on infected RAW 264.7 cells. Infected cells were transferred to a glass slide and air-dried. The cells were first fixed with methanol, then 1 mL of Leishman’s stain was added. After 1 min, the stain was gently diluted by addition of PBS and left for 5 min. The slides were finally rinsed with deionized water and air-dried prior to imaging using the CX33 Olympus light microscope at a magnification of 100× (Shinjuku, Tokyo, Japan).

### 3.13. Statistical Analysis

All experiments were conducted in triplicates (*n* = 3), with results reported as mean ± standard deviation. Experimental data were statistically analyzed using Student’s *t*-test or one-way ANOVA and Tukey’s multiple comparisons test with GraphPad Prism (version 7.03) at a significance level of α = 0.05.

## 4. Conclusions

In this study, an MSNP nanocarrier for the delivery of the antibacterial drug rifampicin was successfully formulated, characterized, investigated for its cellular uptake and finally evaluated for antimicrobial efficacy against intracellular infection. Cellular studies using macrophages provided insights on the importance of size for optimal uptake of MSNP. Rifampicin-loaded MSNP demonstrated superior efficacy compared to free rifampicin. The outcomes of this research support the hypothesis that the use of MSNP as a nanocarrier facilitates the internalization of drug, translating to better efficacy of currently available antibacterial drugs.

The clinical advantage of nanocarrier systems to effectively deliver antibacterial drugs intracellularly may reduce the need for long-term treatment with high doses of the drug, thereby reducing side effects and toxicity. Moreover, this could ultimately reduce the development of resistance towards antibacterial drugs due to minimization of sub-optimal (lethal) concentrations.

With further investigations and successful translation in vivo, MSNP may provide a better delivery platform for antibacterial drugs against intracellular infections, overcoming the barriers of currently available treatment options.

## Figures and Tables

**Table 1:** Particle size and zeta potentials date for MSNP-40 and MSNP-100.

Particles	TEM (nm)	DLS (nm) (Peak Intensity)	PDI	Zeta Potential (mV)
MSNP-40	47.5 ± 4.8	72.9 ± 37.5	0.306 ± 0.01	−20.0 ± 7.36
MSNP-100	78.4 ± 5.7	130.8 ± 53.5	0.587 ± 0.02	−16.9 ± 3.45

**Table 2:** Loading capacity and encapsulation efficiency of rifampicin into MSNP.

Particles	Loading Capacity (% *w*/*w*)	Encapsulation Efficiency (%)
MSNP-Rif 40	38.3	26.8
MSNP-Rif 100	41.1	22.5
